# Interaction Effects of Tannic Acid and Gluten on Bread-Making and Its Starch Digestion

**DOI:** 10.3390/foods14020233

**Published:** 2025-01-13

**Authors:** Seonghyeon Nam, Oguz K. Ozturk, Jongbin Lim

**Affiliations:** 1 Department of Food Bioengineering, Jeju National University, Jeju 63243, Republic of Korea; seonghyeon_n@stu.jejunu.ac.kr; 2 Department of Food Science and Human Nutrition, University of Illinois Urbana-Champaign, Urbana, IL 61801, USA; oozturk3@illinois.edu

**Keywords:** tannic acid, gluten network, baking performance, starch digestion

## Abstract

In this study, we explored the binding mechanism between tannic acid (TA) and gluten to apply TA as an ingredient in bread-making to evaluate its baking performance and starch digestion. The interaction was systematically investigated by analyzing binding affinity, binding mode, and matrix structure of the TA–gluten complex using fluorescence quenching, molecular docking, and confocal laser scanning microscopy. TA strongly interacted with gluten via non-covalent interactions, mainly hydrogen bonds, and formed the major hydrogen bonds with six different glutamines (Q32, Q108, Q313, Q317, Q317, and Q349), which play a critical role in gluten network construction among amino acid residues of gluten. Additionally, TA showed lower binding affinity toward glutenin (−10.4 kcal/mol) compared to gliadin (−8.9 kcal/mol), implying stronger binding with glutenin. Consequently, the interaction between TA and gluten created a dense and compact gluten network structure. It influenced baking performance, causing a decrease in bread loaf volume while an increase in firmness and lowering the starch digestion rate, increasing slowly digestible starch and resistant starch fractions. This study identified the binding mechanism of TA toward gluten and provides better insights into how to apply TA or perhaps other polyphenols to design functional bakery products to control starch digestion rate.

## 1. Introduction

Starch is a major energy source in human nutrition, and it could be nutritionally classified into three distinct fractions based on the digestion rate in the small intestine: rapidly digestible starch (RDS), slowly digestible starch (SDS), and resistant starch (RS) [1]. Particularly, RDS leads to a sharp increase in the postprandial blood glucose levels, and chronic consumption of starch containing high amounts of RDS may initiate hyperglycemia, hyperinsulinemia, and insulin resistance [2]. Thus, considerable attention has been directed towards modulating the digestion rate of carbohydrate-based foods to reduce the risk factors of such chronic diet-related metabolic diseases [3]. Among the most frequently consumed carbohydrate-based foods, bread is a key staple food in the human diet, and many investigations have been conducted to control its starch digestion rate to lower postprandial glycemic response. Reported ways to slow starch digestion rate are to add dietary fiber, such as resistant starch or inulin, in bread-making [4] and to provide a polyphenol-containing beverage as a natural carbohydrate digestive enzyme inhibitor, e.g., lemon and pomegranate juices [5,6], during consumption of bread. Here, our strategy to control the digestion rate of bread was to use plant-based polyphenols as an ingredient in bread-making to induce polyphenol-gluten protein interaction for forming a compact and dense gluten network structure, causing less accessibility of digestive enzymes, endo-acting α-amylase and exo-acting α-glucosidase, to the surface area of starch.

Gluten is composed of monomeric gliadin and polymeric glutenin and forms a continuous and viscoelastic gluten network through hydration and mixing [7]. In the dough system, the gluten matrix encloses starch, and the structure of the gluten network is intimately related to the starch digestion rate of bread. In related work, a low glycemic index of pasta was attributed to entrapped starch in the gluten matrix with the compact and dense microstructure, which acts as a barrier against enzyme access to starch, resulting in slower starch digestion [8]. It has been noted that plant-based polyphenols bind to macronutrients in foods through non-covalent interactions, and, in the case of protein, hydrogen bonding is critically important [9]. Among plant-based phenolic compounds, tannic acid (TA) forms complexes with gluten via multiple hydrogen bonds between the hydroxyl group of TA and the carboxyl group of protein [10]. Additionally, it was reported that TA interacts with wheat gluten via hydrogen bonding as well as hydrophobic interactions, altering the structure and functionality of gluten [11]. Therefore, it is expected that TA can produce binding interactions with gluten, causing a change in its functionality.

Previous studies reported that TA modifies gluten structure [12], and the addition of TA to the bread-making process improves dough mixing properties and retards bread staling [13]. However, the binding mechanism between TA and gluten is relatively unknown. Thus, in this study, we systematically investigated how TA interacts with gluten protein by analyzing binding affinity, binding mode, and matrix structure of the TA–gluten protein complex using fluorescence quenching, molecular docking, and confocal laser scanning microscopy. Furthermore, we explored the effects of TA on baking performance and digestion rate. The results obtained from this study provide insight into how to apply TA or perhaps other polyphenols to develop or design functional bakery products to control starch digestion rate.

## 2. Materials and Methods

### 2.1. Experiment Materials

Tannic acid (TA, Product No.: 403040), gluten from wheat (Product No.: G5004), pancreatin from porcine pancreas (Product No.: P7545), and amyloglucosidase from *Aspergillus niger* (Product No.: A7420) were purchased from Sigma-Aldrich (St. Louis, MO, USA). All ingredients, including wheat flour, salt, sugar, and yeast, were obtained from the local grocery store.

### 2.2. Fluorescent Quenching

Gluten from wheat (10 mg) was mixed with distilled water. TA was mixed with gluten and then incubated at 20 °C for 10 min. The solution was immediately placed into a quartz cuvette cell, and then the fluorescence emission spectra were recorded from 320 to 550 nm of wavelength with 10 nm slits of both excitation and emission at 295 nm using a fluorescence spectrometer (LS55, Perkin-Elmer, Woodbridge, ON, Canada). Quenching parameters (FRET, fluorescence resonance energy transfer; *K*_sv_, quenching constant) were calculated using the following equations:

*FRET* (%) = (*F*_0_ − *F*)/*F*_0_ × 100, (1)

*F*_0_/*F* = 1 + *k*_q_*τ*_0_[*Q*] = 1 + *K*_sv_[*Q*]. (2)

According to Forster’s theory [14], *F*_0_ and *F* are the fluorescence intensities of the gluten in the absence and presence of TA. *K*_sv_ was described by Equation (2), where *k*_q_ is the biomolecular quenching constant, *τ*_0_ is the lifetime of the fluorophore within the protein, and [Q] is the concentration of TA as quencher.

### 2.3. Molecular Modeling

The crystal structure of gliadin (PDB CODE: 4OZF) was obtained from the Protein Data Bank (PDB), and glutenin structure was constructed using the I-TASSER algorithm based on the amino acid sequence obtained from UniProKB (No.: P08488) [15]. The chemical structure of TA was generated using ChemDraw (Perkin Elmer, Waltham, MA, USA). Molecular docking was performed using Autodock Vina (Scripps Research, La Jolla, CA, USA) [16]. The docking output was analyzed using PyMOL (Schrodinger, New York, NY, USA) [17].

### 2.4. Bread-Making

Yeast (0.9 g) was dissolved in 75 mL of pre-warmed water (50 °C), and then transferred into the ingredient mixture containing wheat flour (100 g), sugar (5 g), and salt (1 g). The addition levels of TA were 1, 3, and 5 g. All ingredients were mixed using a mixer (Kitchen Aid, St. Joseph, MI, USA) for 8 min. After mixing, the dough was weighed and then divided into two equal parts. Each part was placed and proofed in a 6-inch mini loaf pan. Dough fermentation was conducted in a fermentation cabinet (InterMetro Industries Co., Wilkes-Barre, PA, USA) at 35 °C for 50 min. It was baked in a rotary electric oven (Doyon Inc., Quebec, QC, Canada) at 163 °C for 30 min and then allowed to cool down at room temperature for 1 h.

### 2.5. Baking Performance

Bread loaf volume was measured by the rapeseed displacement method (AACC Method 10-05.01). Volume was expressed as a percentage (%) compared to the control. For the texture analysis, bread was cut into 25 mm thick slices. The slices were used to determine the hardness (N) of the crumb through a TPA test using a texture analyzer equipped with a 32 mm diameter cylindrical aluminum probe (Stable Micro Systems, Godaiming, UK). The probe penetrated a distance of 15 mm from the surface of the crumb, which represents 60% of the depth of the sample, and the test speed was 1.7 mm/s. A colorimeter (Konica Minolta, Buena Borough, NJ, USA) was used to measure the crust and crumb colors of bread described as L* (lightness/darkness), a* (redness/greenness), and b* (yellowness/blueness) values.

### 2.6. Confocal Laser Scanning Microscopy

Grounded bread samples (20 mg) were mixed with fluorescamine in acetonitrile (200 µL, 0.1%, *w*/*v*) and sodium borate buffer (300 µL, 0.1 M, pH 8). The mixture was reacted at room temperature for 5 min in a dark room. After centrifugation at room temperature and 13,000 rpm for 5 min, the residue was collected and then rinsed with deionized water 5 times. The dried residue was mixed with glycerol solution (200 µL, 75% *w*/*v*), and then aliquots (10 µL) were transferred into microscope slides. A confocal laser scanning microscope (Zeiss LSM 880, Carl Zeiss Microscopy, Oberkochen, Germany) was used to visualize the protein within bread at 405 nm protein excitation, and protein was detected at 450/80 nm.

### 2.7. In Vitro Starch Digestibility

In vitro starch digestibility of bread was measured to determine rapidly digestible starch (RDS) digested within 20 min, slowly digestible starch (SDS) digested between 20 and 120 min, and resistant starch (RS) undigested after 120 min through the previously published method with slight modification [1]. The procedure includes the measurement of free glucose (FG) and total glucose (TG) to calculate each fraction. The amounts of glucose after digestion for 20 min and 120 min are G20 and G120, respectively. The distinct fractions are quantified as follows:

TS = 0.9 × (TG − FG),(3)

RDS = 0.9 × (G20 − FG),(4)

SDS = 0.9 × (G120 − G20),(5)

RS = TS − RDS − SDS.(6)

Bread (2 g) was mixed with water (17.5 mL), and then a solution (10 mL) containing pepsin (5 g/L), HCl (0.05 M/L), and guar gum (pH 2, 5 g/L) was added to simulate gastric conditions and to standardize the viscosity of treatments. The mixture was incubated in a water bath at 37 °C and 160 rpm for 30 min with three glass marbles. After the gastric phases, sodium acetate solution (5 mL, 0.5 mol/L) was added to adjust the pH to 6.9. The enzyme mixture (5 mL) of porcine pancreatin (150 g/L) and amyloglucosidase (40 mL/L) was added and then incubated at 37 °C and 160 rpm for 120 min. Aliquots (100 µL) were removed from the test tube after 20 min and 120 min (also 360 min for an extended digestion process) and then transferred into 900 µL ethanol to terminate the enzyme reaction. The solution was used to quantify RDS, SDS, and RS of bread. Total starch content was measured using a total starch assay kit (K-TSTA-100A, Megazyme, IL, USA). Glucose release was measured using a glucose oxidase/peroxidase (GOPOD) method (K-GLUC, Megazyme, Chicago, IL, USA).

### 2.8. HPLC for Molecular Degradation of Carbohydrate During an In Vitro Digestion Process

After the in vitro digestion process, aliquots (100 µL) were collected from the test tube at 120 min and then immediately transferred into 900 µL of ethanol to determine the molecular degradation. The solutions were centrifuged at room temperature and 5000 rpm for 15 min, and the residues were used to analyze the molecular weight distribution of digesta using an HPLC system equipped with a refractive index (Agilent Technologies, Santa Clara, CA, USA). Injected samples (100 µL), which were filtered through a 0.45 µm nylon syringe filter, were separated on Superdex 30 gel filtration media (GE Healthcare, Chicago, IL, USA). The mobile phase was purified water with 0.02% sodium azide at a flow rate of 0.4 mL/min.

### 2.9. Statistical Analysis

All data were tested in triplicate and were expressed as mean ± SEM. Differences across treatments were analyzed by one-way ANOVA with the post hoc Tukey test using SAS v.9.4 software (SAS Institute, Cary, NC, USA) to form statistical groupings. *p*-values lower than 0.05 were considered significant.

## 3. Results

### 3.1. Binding Affinity of TA Toward Gluten Protein

The binding affinity between a ligand and protein has been widely studied through intrinsic tryptophan fluorescence quenching [18], and many researchers have reported that polyphenols interact with gluten protein [19]. The fluorescence quenching technique was applied to investigate the binding affinity of tannic acid (TA), which consists of a central glucose linked with multiple galloyl groups (Supplementary Figure S1), towards gluten protein.

**Figure 1:**
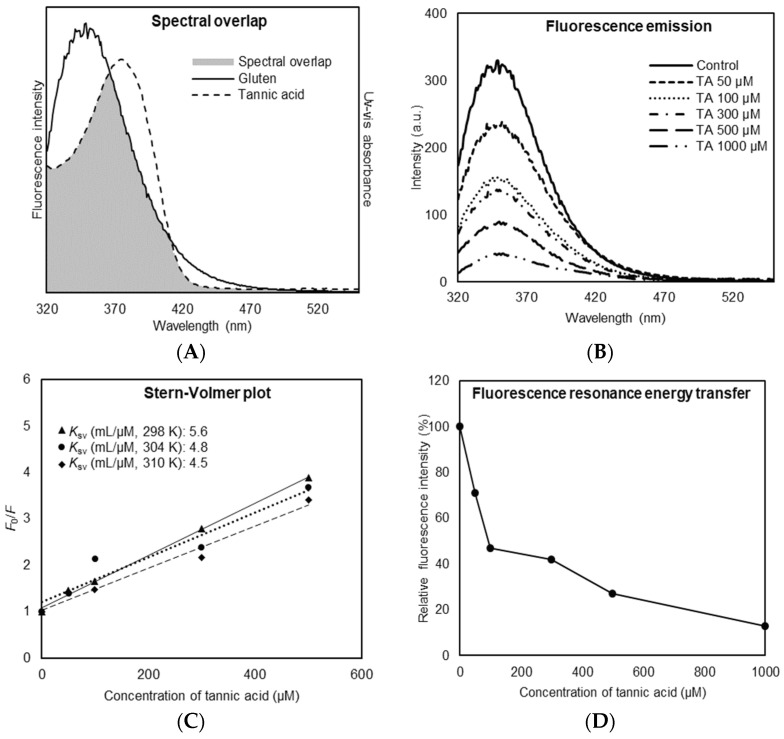
Binding affinity between tannic acid (TA) and gluten protein. (**A**) Spectral overlap between UV–Vis absorbance of TA and fluorescence intensity of gluten. (**B**) Fluorescence emission spectra of gluten in the presence of TA at different concentrations (50/100/300/500/1000 µM). (**C**) Stern–Volmer plot for fluorescence quenching of gluten in the presence of TA at different concentrations (50/100/300/500/1000 µM). (**D**) Relative fluorescence intensity of gluten at different concentrations of TA (50/100/300/500/1000 µM).

The fluorescence emission spectra of gluten protein and the UV–Vis absorbance spectra of TA were measured and showed integral overlapping area (Figure 1A). The overlapping area indicates that TA causes intrinsic tryptophan fluorescence quenching and binds to gluten protein [14,20]. Furthermore, others have shown that the quenching occurs via non-radiative energy transfer and that fluorescence resonance energy is transferred from the protein (i.e., gluten) as a donor to the ligand (i.e., TA) as an acceptor [21]. As shown in Figure 1B, fluorescence emission spectra of the gluten protein were gradually reduced by increasing the concentration of TA. There was a little shift in the fluorescence peak intensity from 348 to 350 nm with the addition of TA in the range of concentrations from 50 to 1000 µM. This implies that TA was closely located to the tryptophan residue for the quenching to occur, and there was a conformational change in gluten protein structure due to TA binding [22]. Figure 1C shows linear Stern–Volmer plots constructed by the fluorescence intensity of gluten protein quenched by TA at the different temperatures. An increase in temperature from 298 to 310 K resulted in a decrease in the Stern–Volmer quenching constant (*K*_sv_), indicating an inverse correlation. It demonstrated that the fluorescence quenching process is initiated by the formation of a ground state complex between TA and gluten protein, which is referred to as a static quenching mechanism [23,24]. Figure 1D shows a decrease in fluorescence intensity of gluten protein quenched by TA at the different concentrations from 50 to 1000 µM. The addition of TA eventually led to a large reduction in fluorescence intensity, having 72.8% fluorescence energy transfer (FRET), in which energy transfer must be passed non-radiatively between the TA and gluten protein. It suggests that TA has a remarkable quenching capacity and considerable binding property toward gluten protein.

### 3.2. Binding Mode of TA Toward Gluten Protein

Gluten consists of two storage proteins, gliadin and glutenin, and the two proteins form a continuous and viscoelastic gluten network through inter- and intra-molecular SS bonds. The protein group has its distinct functionality in the formation of the gluten network, as gliadin contributes mainly to the extensibility and viscosity, whereas glutenin is primarily responsible for the elasticity and strength [25]. In this section, molecular modeling using Autodock Vina was used to investigate the binding mechanism (i.e., binding affinity and hydrogen network) of TA towards the two gluten proteins [16].

**Figure 2:**
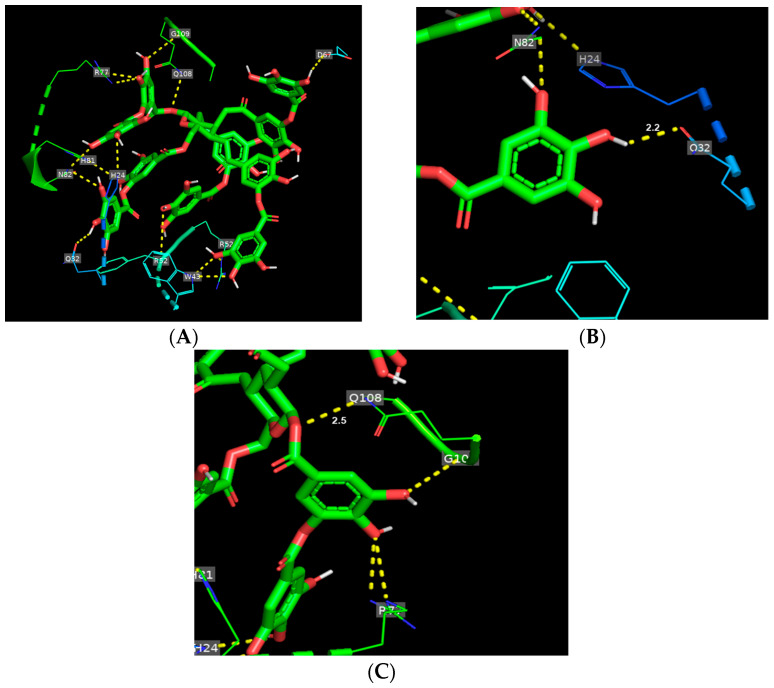
Binding mode of tannic acid (TA) toward gliadin. (**A**) Schematic representation of hydrogen bindings (yellow dashed line) between TA (green) and amino acid residues of gliadin. (**B**,**C**) Main hydrogen bonds between TA and glutamines (Q32 and Q108) of gliadin.

**Figure 3:**
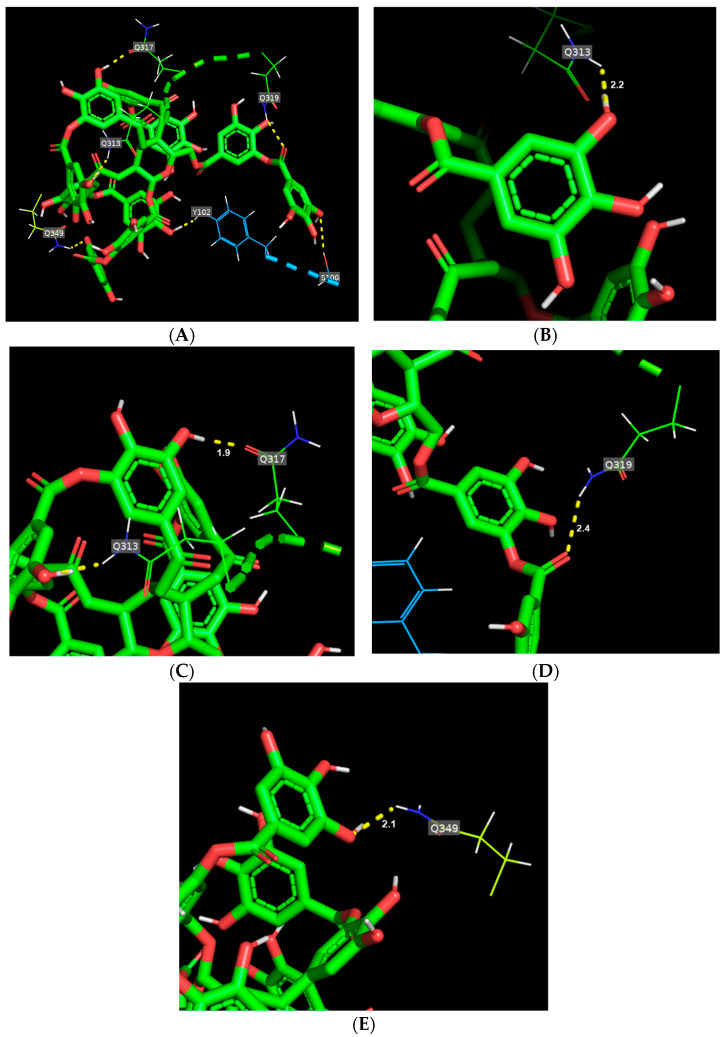
Binding mode of tannic acid (TA) toward glutenin. (**A**) Schematic representation of hydrogen bonds (yellow dashed line) between TA (green) and amino acid residues of glutenin. (**B**–**E**) main hydrogen bonds between TA and glutamines (Q313, Q317, Q319, and Q349) of glutenin.

Figure 2A shows the binding mode of TA toward gliadin. TA interacted with 10 amino acid residues (D67, G109, H24, H81, Q32, Q108, R52, R77, and W43) of gliadin, inducing a total of 14 hydrogen bonds. In the hydrogen bond network, TA is bound tightly to two glutamines, Q32 and Q108, having shorter hydrogen bond distances of 2.2 and 2.5, respectively (Figure 2B,C, Supplementary Table S1). For glutenin, TA formed six hydrogen bonds with six different amino acid residues (Figure 3A and Supplementary Table S1) and mainly interacted with four glutamines that were from 1.9 to 2.4, with TA maintaining the shortest hydrogen bond distance with Q317 among the six hydrogen bonds (Figure 3C and Supplementary Table S1).

Glutamine is the most abundant amino acid of gluten protein, making up 36% of the total amino acids [26]. Glutamine produces numerous inter-chain hydrogen bonds with other glutamines as well as hydroxyl amino acids (i.e., serine and tyrosine). The individual hydrogen bonds are weak, but collectively they create a strong gluten network structure [27]. A strong gluten network retains more gas than a weak gluten network and produces a higher bread loaf volume [28].

TA showed a lower binding affinity for glutenin (−10.4 kcal/mol) compared to gliadin (−8.9 kcal/mol), implying stronger binding with glutenin than gliadin (Supplementary Table S2). Since polyphenols have distinct binding affinities towards proteins based on their chemical ring structure [29,30,31], the application of various polyphenols could cause different gluten network structures through distinct interactions with gliadin and glutenin. Therefore, a further understanding of the structural specificity of polyphenols towards gluten protein is needed to manipulate gluten network structure for obtaining adequate bread quality with a lowered postprandial glycemic response.

### 3.3. Baking Performance of TA-Treated Bread

As described above, TA binds to gluten protein mainly via hydrogen bonds. The interaction implies that TA induces a change in gluten network structure, leading to an influence on bread baking performance. Thus, the effects of TA on bread baking performance were investigated by measuring its volume, texture, and color.

**Figure 4:**
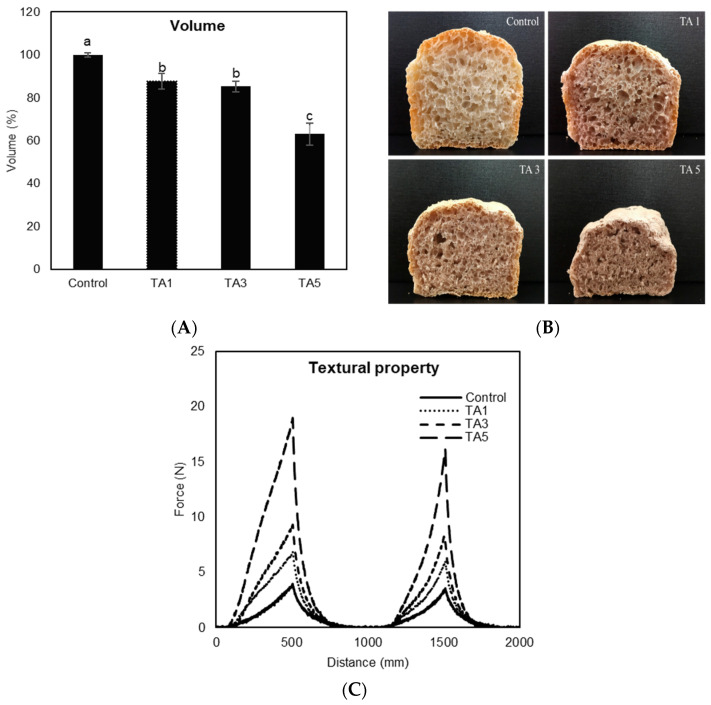
Effect of tannic acid (TA) on baking performance. (**A**) Bread loaf volume. Different letters on bars indicate a significant difference at the 5% level. (**B**) Visual appearance of sliced bread loaf. (**C**) Textural property of bread.

**Figure 5:**
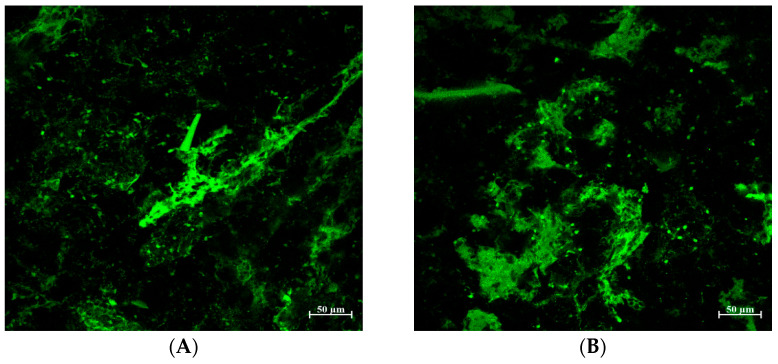

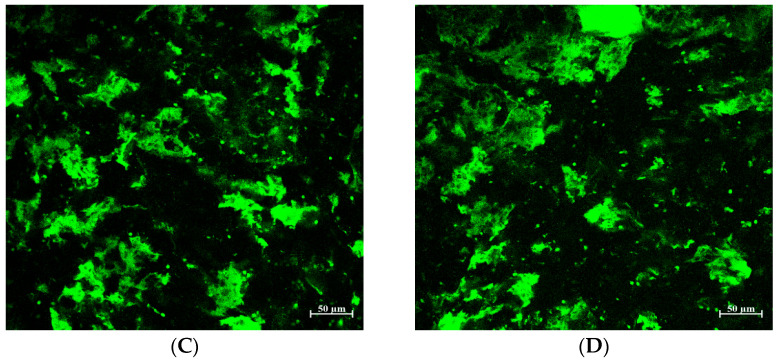
Gluten matrix structure of bread treated with tannic acid (TA). (**A**) Control (**B**) TA1 (**C**) TA5 (**D**) TA5.

Application of TA to bread-making caused a significant decrease in bread loaf volume (*p* < 0.05), with incremental decreases up to 36.8% compared to the control, with increasing the concentration of TA (Figure 4A). Control has significantly higher volume than all TA treatments, and the volumes of TA1 and TA3 are not significantly different. TA5 showed significantly lower volume than all treatments. Figure 4B shows the visual appearance of sliced bread loaves. The sliced sections treated with TA were expectedly smaller than the control, with the addition of 5% TA having the smallest size loaf. Furthermore, the slices from TA-containing breads had comparatively smaller pores on the surface. The decrease is reportedly due to the chemical reaction of TA acting as a redox agent during a gluten network formation process, causing a structural change in the gluten network [30]. Gluten is connected by inter- and intra-molecular disulfide (SS) bonds to form strong cross-links between polypeptide chains, producing a viscoelastic and continuous gluten network that entraps carbon dioxide (CO_2_) gas to give bread its volume [7]. Since the free radicals of TA react with SS bonds of gluten proteins, TA cleaves SS bonds between gluten proteins and induces sulfhydryl/disulfide (SH/SS) bond exchange reactions yielding a lesser amount of SS bonds [13]. Consequently, it leads to a dense and compact gluten network structure accompanied by poor gas retention, resulting in a low bread loaf volume as well as smaller pores in the crumb structure. Texture profile analysis (TPA) test, commonly referred to as the two-bite test, was applied to measure the textural property of bread treated with TA (Figure 4C). Bread hardness, which corresponds to the maximum force during the first compression, was significantly and gradually increased as more TA was used (*p* < 0.05), with the increased hardness value of breads increasing from 4.0 to 19.0 N for the control and 5% TA treatment, respectively. It is likely the result of poor gas retention within the gluten network by the addition of TA to bread-making, resulting in a compact and dense structure of bread. The color of the bread crust and crumb is shown in Table 1. As more TA was applied, the L value of the crust significantly increased (*p* < 0.05), indicating more lightness, while the bread crumb became darker in color with reduced L values (*p* < 0.05), as shown in Figure 5B. The use of TA produced a considerable change in the a value, which indicates the green/red coordinate of crust and crumb (*p* < 0.05), showing greener on the crust but redder on the crumb than the control. Also, the addition of TA caused a significant decrease in the b value of crust and crumb (*p* < 0.05), indicating more bluish color compared to the control. Overall, the total color difference (DE) of bread crust and crumb was significantly dependent on the amount of TA (*p* < 0.05).

Among various bread-making properties, loaf volume, texture or firmness, and color dramatically influence consumer perception of bread quality [32]. In this study, the addition of TA to bread formulations induced significant changes in the key attributes of bread, causing a decrease in loaf volume and an increase in firmness. Even though previous studies have reported that the application of dietary polyphenols to bread-making enhances several intrinsic bread-making features such as freshness and dough quality [13,33], more applied formulation research is needed, such as fine-tuning TA concentration to obtain lower postprandial glycemia while maintaining adequate bread quality.

### 3.4. Protein Structure in Bread Treated with TA

As mentioned, the addition of TA to the bread formulation produced a dense and compact bread structure represented by low loaf volume and a firmer texture compared to the control. To better understand the effect of TA on protein matrix structure, confocal laser scanning microscopy (CLSM) was used with fluorescamine for protein-specific dyeing (Figure 5, proteins appear as green) [34]. Additionally, breads treated with TA at different concentrations had differently appearing protein matrices compared to the control.

In the control bread (Figure 5A), a fine-stranded gluten network is clearly recognizable. On the other hand, breads treated with different levels of TA exhibited increasingly discontinuous matrices and dense-appearing aggregates rather than the fibrous matrix of the control bread (Figure 5B–D). The dense and tight protein aggregates in the micrographs of breads treated with TA explain the lack of air cells that are necessary to retain the gasses to rise in bread volume, which supports the observed low loaf volume and increased firmness compared to the control (Figure 4).

### 3.5. Starch Digestion Property of TA-Treated Bread

The finding that TA interacts closely with gluten protein, producing a dense and compact structure of the gluten network as well as a firm texture, implies lower accessibility of starch digestive enzymes to starch, causing a slower digestion rate. The hydrolysis property of TA-treated bread during a digestion process was analyzed using the in vitro assay and HPLC system to evaluate its digestion rate.

**Figure 6:**
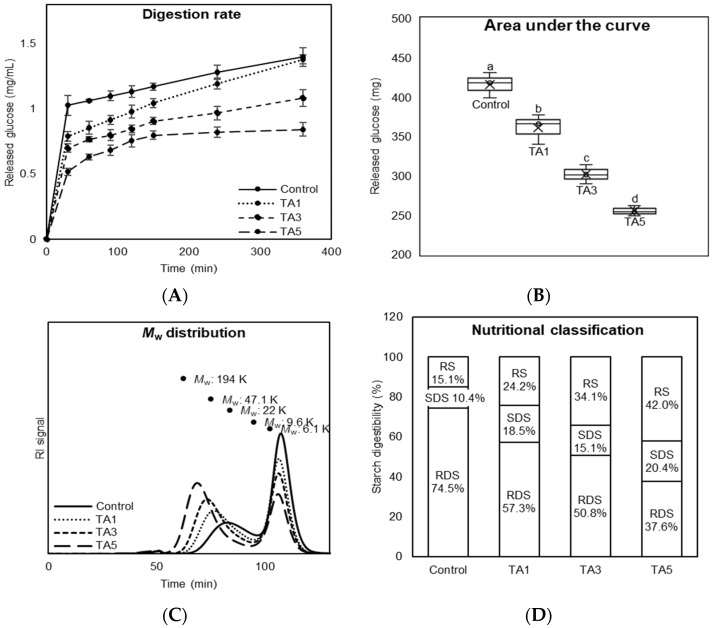
Digestion property of bread treated with tannic acid (TA). (**A**) Digestion rate of bread by starch digestive enzymes at 37 °C for 6 h. (**B**) Area under the curve (AUC) of total released glucose amount during a 6 h digestion process. Different letters on bars indicate a significant difference at the 5% level. (**C**) Molecular weight distribution of the remaining starch fractions from in vitro digestion of bread. (**D**) Nutritional classification of bread.

Figure 6A shows the amount of glucose released from TA-treated bread during an extended 6 h digestion process. The application of TA in bread-making reduced glucose release compared to the control, with more reduction with increasing TA concentration. Furthermore, the area under the curve (AUC) of total released glucose amount was significantly lower for TA-treated breads than the control (*p* < 0.05), and the amount was dramatically decreased up to 61.3% in the TA5 treatment (Figure 6B). Figure 6C indicates the molecular weight (*M*_w_) distribution of the undigested starch and digested starch products (i.e., glucose) of bread at the 120 min post-digestion. In the chromatograms, TA treatments had higher *M*_w_ distribution than the control, indicating less digestion as confirmed in the results of glucose release (Figure 6A). Additionally, the TA5 treatment had the lowest second peak (predominantly glucose) among the treatments, convincingly supporting the lowest released glucose amount of the TA5 treatment (Figure 6B). Figure 6D shows the nutritional classification of bread treated with TA based on the in vitro digestion rate. Starch is classified into three nutritional fractions based on its digestibility: rapidly digestible starch (RDS), slowly digestible starch (SDS), and resistant starch (RS) [1]. The addition of TA to bread-making induced a significant reduction in the amount of RDS of up to 36.9% compared to the control, while the amount of SDS was increased by up to 10%. The amount of RS was substantially increased up to 26.9% with increasing the concentration of TA. The increased amount of SDS has a considerable potential to increase the secretion of Glucagon-like peptide-1 (GLP-1) hormone, which plays a critical role in controlling satiety or eating habits, from enteroendocrine L-cells in the distal small intestine [35]. The previous study showed that an increase in GLP-1 secretion from slowly digestible starch deposited in the distal small intestine reduced food intake and body weight gain in a 6-week mouse feeding study [36]. It means that TA-treated bread with higher SDS could be considered as a promising new strategy to manage food intake as well as weight gain.

Overall, the application of TA to bread-making slowed its digestion rate, and the slow digestion improved the nutritional quality of bread, increasing SDS and RS, as well as decreasing RDS. Even though it has been reported that TA has the inhibition property against starch digestive enzymes, the bread treated with TA showed a negligible inhibition property due to the loss of antioxidant activity during the heating process. Consequently, it is likely the result of the interaction between TA and gluten protein, forming the compact and dense gluten network structure, which allows accessibility of starch digestive enzymes to starch.

## 4. Conclusions

In this study, the interaction between TA and gluten protein was investigated by fluorescence quenching, molecular docking, and confocal laser scanning microscopy. TA interacted with gluten protein mainly via hydrogen bonds and had binding with six glutamines (Q32, Q108, Q313, Q317, Q319, and Q349) among the amino acid residues of gliadin and glutenin, creating a dense and compact gluten network structure. Consequently, the dense and compact gluten network structure induced lower bread loaf volume and a harder texture and improved nutritional quality by increasing SDS and RS fractions while decreasing the RDS portion. Therefore, the application of TA or other polyphenols as an ingredient in bread-making can be considered a promising approach for designing functional bakery products to control their digestion rate. A further understanding is required of the structural specificity of polyphenols to manipulate gluten network structure to obtain adequate bread quality as well as a slow digestion rate.

## Figures and Tables

**Table 1:** Color change in the crust and crumb of bread treated with tannic acid (TA).

Color		Control	TA1	TA3	TA5
Crust	L	66.48 ^b^	67.01 ^b^	67.11 ^b^	75.35 ^a^
	a	9.63 ^a^	9.01 ^a,b^	8.77 ^b^	4.94 ^c^
	b	28.00 ^a^	27.04 ^a^	25.69 ^b^	16.47 ^c^
	DE ^A^		1.26 ^c^	2.54 ^b^	15.28 ^a^
Crumb	L	67.11 ^a^	56.74 ^c^	60.65 ^b^	61.60 ^b^
	a	0.64 ^b^	7.50 ^a^	7.76 ^a^	7.81 ^a^
	b	19.87 ^a^	14.06 ^b^	14.18 ^b^	13.99 ^b^
	DE ^A^		10.53 ^b^	11.16 ^b^	19.90 ^a^

Values in the same row with different superscript lowercase indicate significant difference (*p* < 0.05). ^A^ Total color difference.

## Data Availability

The original contributions presented in the study are included in the article/Supplementary Materials, further inquiries can be directed to the corresponding author.

## Supplementary Materials

The following supporting information can be downloaded at https://www.mdpi.com/article/10.3390/foods14020233/s1, Figure S1: Chemical structure of tannic acid (TA); Table S1: Distance of hydrogen bond between amino acid residues of gluten proteins and tannic acid (TA); Table S2: Binding affinity of tannic acid (TA) towards gluten proteins.

## References

[1] Englyst H.N., Kingman S., Cummings J. (1992). Classification and measurement of nutritionally important starch fractions. Eur. J. Clin. Nutr..

[2] Englyst H.N., Veenstra J., Hudson G.J. (1996). Measurement of rapidly available glucose (RAG) in plant foods: A potential in vitro predictor of the glycemic response. Br. J. Nutr..

[3] Wang Y., Zhou X., Xiang X., Miao M. (2023). Association of slowly digetible starch intake with reduction of postprandial glycemic response: An update meta-analysis. Foods.

[4] Sciarini L.S., Bustos M., Vignola M.B., Paesani C., Salinas C., Perez G.T. (2017). A study on fibre addition to gluten free bread: Its effects on bread quality and in vitro digestibility. J. Food Sci. Technol..

[5] Freitas D., Boué F., Benallaoua M., Airinei G., Benamouzig R., Le Feunteun S. (2021). Lemon juice, but not tea, reduces the glycemic response to bread in healthy volunteers: A randomized crossover trial. Eur. J. Nutr..

[6] Kerimi A., Nyambe-Silavwe H., Gauer J.S., Tomás-Barberán F.A., Williamson G. (2017). Pomegranate juice, but not an extract, confers a lower glycemic response on a high–glycemic index food: Randomized, crossover, controlled trials in healthy subjects. Am. J. Clin. Nutr..

[7] Wieser H. (2007). Chemistry of gluten proteins. Food Microbiol..

[8] Zou W., Sissons M., Gidley M.J., Gilbert R.G., Warren F.J. (2015). Combined techniques for characterising pasta structure reveals how the gluten network slows enzymic digestion rate. Food Chem..

[9] Bordenave N., Hamaker B.R., Ferruzzi M.G. (2014). Nature and consequences of non-covalent interactions between flavonoids and macronutrients in foods. Food Funct..

[10] Hager A.-S., Vallons K.J., Arendt E.K. (2012). Influence of gallic acid and tannic acid on the mechanical and barrier properties of wheat gluten films. J. Agric. Food Chem..

[11] Girard A.L., Awika J.M. (2020). Effects of edible plant polyphenols on gluten protein functionality and potential applications of polyphenol–gluten interactions. Compr. Rev. Food Sci. Food Saf..

[12] Girard A.L., Bean S.R., Tilley M., Adrianos S.L., Awika J.M. (2018). Interaction mechanisms of condensed tannins (proanthocyanidins) with wheat gluten proteins. Food Chem..

[13] Zhang L., Cheng L., Jiang L., Wang Y., Yang G., He G. (2010). Effects of tannic acid on gluten protein structure, dough properties and bread quality of Chinese wheat. J. Sci. Food Agric..

[14] Sahoo H. (2011). Förster resonance energy transfer–A spectroscopic nanoruler: Principle and applications. J. Photochem. Photobiol. C Photochem. Rev..

[15] Yang J., Yan R., Roy A., Xu D., Poisson J., Zhang Y. (2015). The I-TASSER Suite: Protein structure and function prediction. Nat. Methods.

[16] Trott O., Olson A.J. (2010). AutoDock Vina: Improving the speed and accuracy of docking with a new scoring function, efficient optimization, and multithreading. J. Comput. Chem..

[17] Seeliger D., de Groot B.L. (2010). Ligand docking and binding site analysis with PyMOL and Autodock/Vina. J. Comput.-Aided Mol. Des..

[18] Eftink M.R., Ghiron C.A. (1981). Fluorescence quenching studies with proteins. Anal. Biochem..

[19] Kan L., Capuano E., Fogliano V., Oliviero T., Verkerk R. (2020). Tea polyphenols as a strategy to control starch digestion in bread: The effects of polyphenol type and gluten. Food Funct..

[20] Wang J., Wei J., Su S., Qiu J. (2015). Novel fluorescence resonance energy transfer optical sensors for vitamin B 12 detection using thermally reduced carbon dots. New J. Chem..

[21] Khan S.N., Islam B., Yennamalli R., Sultan A., Subbarao N., Khan A.U. (2008). Interaction of mitoxantrone with human serum albumin: Spectroscopic and molecular modeling studies. Eur. J. Pharm. Sci..

[22] Soares S., Mateus N., De Freitas V. (2007). Interaction of different polyphenols with bovine serum albumin (BSA) and human salivary α-amylase (HSA) by fluorescence quenching. J. Agric. Food Chem..

[23] Yan J., Zhang G., Pan J., Wang Y. (2014). α-Glucosidase inhibition by luteolin: Kinetics, interaction and molecular docking. Int. J. Biol. Macromol..

[24] Zhang G., Ma Y., Wang L., Zhang Y., Zhou J. (2012). Multispectroscopic studies on the interaction of maltol, a food additive, with bovine serum albumin. Food Chem..

[25] Lagrain B., Thewissen B.G., Brijs K., Delcour J.A. (2008). Mechanism of gliadin–glutenin cross-linking during hydrothermal treatment. Food Chem..

[26] Wang Y., Gan J., Zhou Y., Cheng Y., Nirasawa S. (2017). Improving solubility and emulsifying property of wheat gluten by deamidation with four different acids: Effect of replacement of folded conformation by extended structure. Food Hydrocoll..

[27] Hoseney R.C. (1994). Principles of Cereal Science and Technology.

[28] Sim S., Aziah A.N., Cheng L. (2015). Quality and functionality of Chinese steamed bread and dough added with selected non-starch polysaccharides. J. Food Sci. Technol..

[29] Lim J., Ferruzzi M.G., Hamaker B.R. (2022). Structural requirements of flavonoids for the selective inhibition of α-amylase versus α-glucosidase. Food Chem..

[30] Yuan W., Fan W., Mu Y., Meng D., Yan Z., Li Y., Lv Z. (2021). Baking intervention for the interaction behaviours between bamboo (Phyllostachys heterocycla) leaf flavonoids and gliadin. Ind. Crop. Prod..

[31] Lim J., Zhang X., Ferruzzi M.G., Hamaker B.R. (2019). Starch digested product analysis by HPAEC reveals structural specificity of flavonoids in the inhibition of mammalian α-amylase and α-glucosidases. Food Chem..

[32] Gellynck X., Kühne B., Van Bockstaele F., Van de Walle D., Dewettinck K. (2009). Consumer perception of bread quality. Appetite.

[33] Wang Q., Li Y., Sun F., Li X., Wang P., Sun J., Zeng J., Wang C., Hu W., Chang J. (2015). Tannins improve dough mixing properties through affecting physicochemical and structural properties of wheat gluten proteins. Food Res. Int..

[34] Ozturk O.K., Kaasgaard S.G., Palmén L.G., Vidal B., Hamaker B.R. (2021). Protein matrix retains most starch granules within corn fiber from corn wet-milling process. Ind. Crop. Prod..

[35] Baggio L.L., Drucker D.J. (2007). Biology of incretins: GLP-1 and GIP. Gastroenterology.

[36] Lim J., Ferruzzi M.G., Hamaker B.R. (2021). Dietary starch is weight reducing when distally digested in the small intestine. Carbohydr. Polym..

