# Supplementary material for: Interaction Effects of Tannic Acid and Gluten on Bread-Making and Its Starch Digestion
Source: Foods. 2025 Jan 13;14(2):233. doi: 10.3390/foods14020233 (PMC11765497; doi:10.3390/foods14020233)
Supplement: Supplementary file 1 [file foods-14-00233-s001.zip › foods-3319307-supplementary.pdf]

Supplementary Figure S1. Chemical structure of tannic acid (TA).

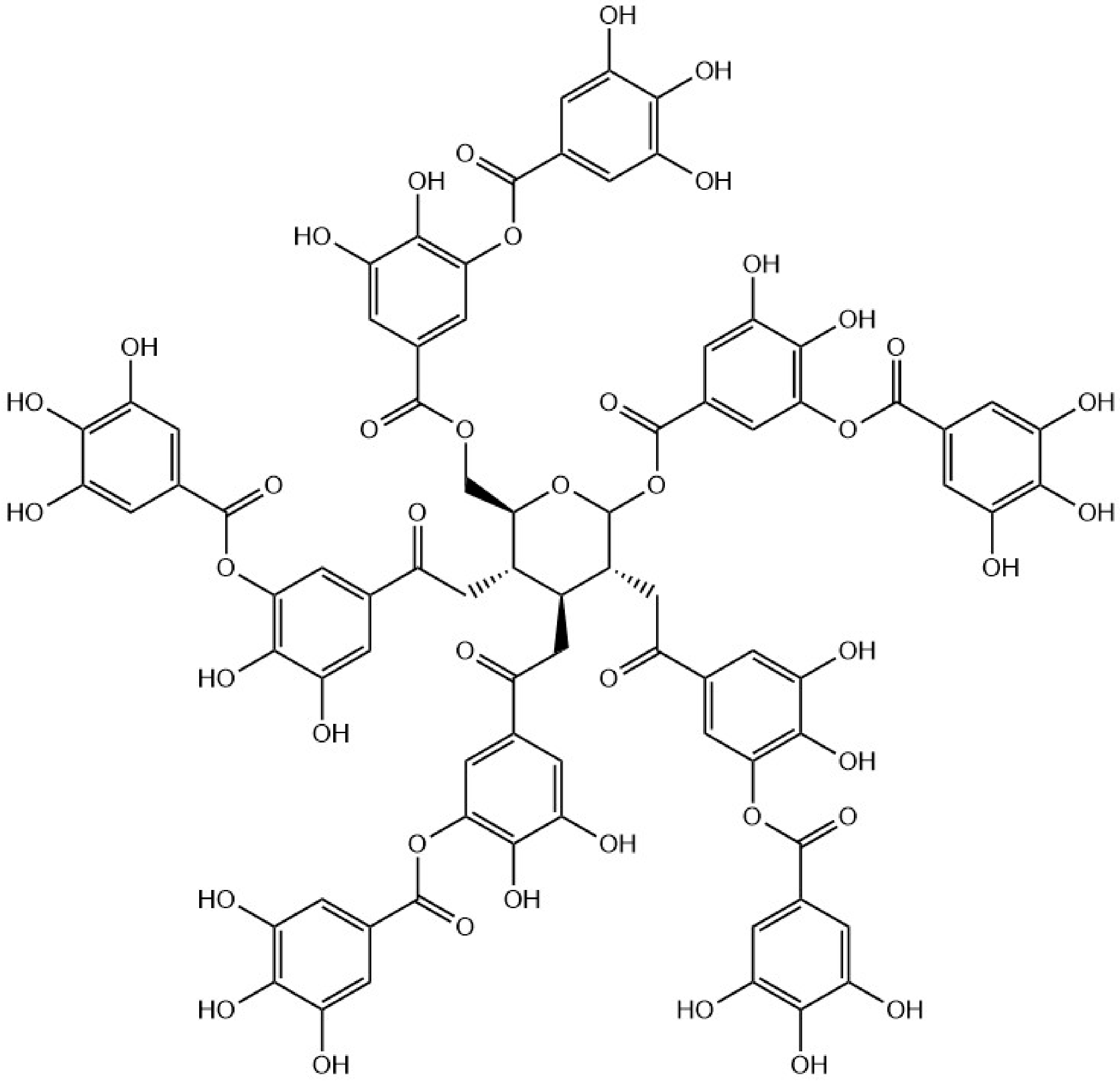

Supplementary Table S1. Distance of hydrogen bond between amino acid residues of gluten proteins and tannic acid (TA).

Protein	Amino acid	Distance
Gliadin	D67	2.6
	G109	3.7
	H24	3.2
	H81	3.3
	N82	3.1
	N82	3.0
	Q32	2.2
	Q108	2.5
	R52	3.0
	R52	4.3
	R77	3.4
	R77	3.2
	W43	3.1
	W43	3.0
Glutenin	Q317	1.9
	Q319	2.4
	Q313	2.2
	Q349	2.1
	S100	2.9
	Y102	2.2

Supplementary Table S2. Binding affinity of tannic acid (TA) towards gluten proteins.

	**Binding Affinity (kcal/mol)**
Gliadin	−8.9
Glutenin	−10.4
